# General Practice Nurses' experiences of changing care delivery during COVID‐19. Implications for future practice: Qualitative study protocol

**DOI:** 10.1111/jan.15312

**Published:** 2022-05-27

**Authors:** Helen Anderson, Arabella Scantlebury, Paul Galdas, Joy Adamson

**Affiliations:** ^1^ York Trials Unit, Department of Health Sciences University of York York UK; ^2^ Department of Health Sciences University of York York UK

**Keywords:** Covid‐19, general practice, general practice, NASSS framework, nursing, nursing change implementation, primary care, qualitative research, remote consultations, study protocol

## Abstract

**Aim:**

- To explore how General Practice Nurses experience implementing change at pace and scale in delivering care during consecutive waves of the COVID‐19 pandemic.
- To evaluate the impact of changes to general practice nurses' working practices on professional wellbeing.

**Background:**

In response to the COVID‐19 pandemic, general practice rapidly and extensively changed care delivery. There has been little exploration of the experiences of General Practice Nurses and care delivery, job satisfaction, workload, stress and professional support.

**Design:**

A qualitative case study design of three to five general practice case sites will explore General Practice Nurses' experiences during the Covid‐19 pandemic. The study was funded and approved by the General Nursing Council Trust in June 2021. University ethics approval was gained in July 2021. Health Research Authority approval has been obtained [IRAS:30353. Protocol number: R23982. Ref 21/HRA/5132. CPMS: 51834].

**Methods:**

Data will consist of focus groups and/or semi‐structured interviews with General Practice Nurses, primary healthcare team members and other key informants. Business/strategy and nurse team meetings relating to workforce planning/review will be observed. Documents will be analysed and routinely collected general practice data will provide descriptive contextualisation at each site. The study will be theoretically underpinned by the Non‐adoption, Abandonment, Scale‐up, Spread and Sustainability Framework and data analysed using framework analysis.

**Discussion:**

General Practice Nurses have a unique sphere of knowledge and undertake specific work in primary care. This workforce is challenged by recruitment, retention and retirement issues, leading to the loss of highly experienced and knowledgeable professionals. It is important to explore how working practices brought about by Covid‐19 affect General Practice Nurses.

**Impact:**

This study will explore working practices brought about by the Covid‐19 pandemic to inform care delivery, patient care and support General Practice Nursing workforce wellbeing and will highlight and mitigate negative aspects of novel and changing care delivery. Key factors in implementing and supporting future practice and change implementation will be developed.

Trial registration: CPMS: 51834.

## 1 INTRODUCTION

Delivery of primary care has changed significantly since the start of the COVID‐19 pandemic in early 2020, both in England (Mroz et al., 2020) and internationally (Rawaf et al., 2020; Verhoeven et al., 2020; Wherton et al., 2020). Changing care delivery and implementing new ways of working have the potential to enhance and improve patient care and healthcare delivery in general practice. However, such implementation requires a robust evidence base and little attention has been focused on the work and experiences of General Practice Nurses [GPN] throughout this time. This is significant as GPNs make a distinct and significant contribution to patient care. The GPN workforce is central to general practice healthcare delivery, provides an interface between health and social care and provides specific care not delivered by others.

This study will identify aspects of patient care and workforce practices brought about by the pandemic to inform and enhance future practice, GPN education and patient care. It is also intended to highlight potential difficulties and negative aspects of implementation. Key factors for success in implementing and supporting different ways of working for GPNs will be identified. Working practices and systems which develop and streamline care delivery, improve patient care and support the wellbeing of the primary care workforce will be highlighted. The study will also identify the scope for the development of GPN education through identifying skills and knowledge required to deliver new and changing ways of working. Identifying factors which may support the GPN workforce has the potential to positively impact on patient care.

## 2 BACKGROUND

In response to the COVID‐19 pandemic, ‘lockdown’ restrictions and UK (and devolved) Government directives, general practice rapidly and extensively changed working practices and care delivery from March 2020 onwards (Mroz et al., 2020). This is reflected internationally (Rawaf et al., 2020; Verhoeven et al., 2020; Wherton et al., 2020). Patient consultations changed from mainly face‐to‐face to almost exclusively remote (71%–89%) including telephone (61%), online (4%) and SMS/email (6%; Mroz et al., 2020: RCGP, 2020; QNI, 2020). However, while novel consultation methods have been demonstrated to be safe and effective (Wherton et al., 2020), studies were often underpowered and not designed to evaluate the management of acute/serious illness (Greenhalgh et al., 2020). Potential wider consequences of remote working on practice populations are also unclear. However, the pandemic has also provided a catalyst for changes which, while identified as potentially beneficial to patients, were previously considered difficult to implement and upscale (Wherton et al., 2020). It has also allowed more flexible working for healthcare professionals (Khan et al., 2020) and improved access for some patients.

General Practice in England, and in other countries, has responded to the pandemic in other ways. Triage has been expanded, work deemed non‐essential postponed and essential work carried out differently (Verhoeven et al., 2020). In England, General Practices (and staff) also underwent rapid reorganization to operate hubs across Primary Care Networks (PCNs) separating those suspected of having COVID‐19 from non‐COVID‐19 care (Khan et al., 2020). Suspended non‐urgent secondary care provision also impacted on primary care providers (Verhoeven et al., 2020). Furthermore, only a small proportion of those diagnosed with COVID‐19 are managed in secondary care, with primary care delivering the bulk of service provision and dealing with ‘collateral damage’ (Rawaf et al., 2020; p. 130) caused by resource diversion and reduced access (Khan et al., 2020). Face‐to‐face primary care access was also initially impacted by lack of personal protective equipment (PPE) (Rawaf et al., 2020). Latterly, general practices have responded at speed to the large‐scale roll out of COVID‐19 vaccinations. While the longstanding effects of COVID‐19 remain unclear, it is anticipated primary care will be central in managing long‐term sequelae.

General Practitioners' [GPs] experiences have been captured during this time (Gray et al., 2020; Khan et al., 2020; RCGP, 2020; Verhoeven et al., 2020), and potential implications for future practice explored. However, there has been little exploration of the experiences of General Practice Nurses (GPNs) in adapting their practice, or consideration of how such rapid and extensive change has impacted on care delivery, job satisfaction, workload, stress, burnout and professional support. A Queen's Nursing Institute (QNI, 2020) survey indicated that, exacerbated by the pandemic, GPNs felt undervalued and this was reflected in poor employment terms and conditions, including remuneration. They also felt GP colleagues deflected face‐to‐face consultations onto GPNs. Indeed, Murphy et al. (2021) showed that 90% of GP consultations were held remotely between April–July 2020, compared with 46% for GPNs. Crucially, the QNI survey showed lack of respect, support and poor employment conditions led some GPNs to consider leaving their position. This is significant because the GPN workforce has experienced recruitment and retention issues long before the pandemic, with a significant ‘retirement bubble’ leading to loss of a highly experienced and knowledgeable workforce which are difficult to replace (HEE, 2017). GPNs have a unique sphere of knowledge and undertake specific work different to that of other primary healthcare professionals. For example, GPNs deliver the bulk of long‐term condition management, essential care which cannot be delayed, public health interventions such as childhood immunization and other vaccination programmes (PHE, 2020), mental health support and procedures requiring face‐to‐face consultations (e.g. complex dressings and cervical cytology).

Whilst the Royal College of Nursing (RCN) has produced general COVID‐19 guidance for nurses (RCN, 2020) the practicalities of rapid and extensive change in GPN service delivery are unclear. For example, how should GPNs effectively teach individuals to give themselves injections, change dressings or check their own blood pressure remotely? How are ethical issues, safeguarding and confidentiality managed? How are childhood immunisations safely administered? How are new guidelines rapidly translated and implemented into nursing practice? What issues are experienced by GPNs delivering face‐to‐face patient care? GPN leaders argue GPNs should take ownership of their sphere of general practice to ensure a post‐COVID future uses and values their skills and knowledge to enhance patient care (Massey, 2020). However, experiences of GPNs throughout COVID‐19 have not been explicitly researched in‐depth. By gaining insight into the development of GPN practice triggered by the pandemic, new ways of working can be identified and evaluated, and barriers and facilitators highlighted. The current study asks how GPNs enact, experience and perceive their roles during the Covid‐19 pandemic to:

- explore changes to GPN practice and care delivery, including technology‐medicated working,
- find out what works well and how practice could be improved, and
- gain insight into how changes in care delivery during the pandemic may affect GPN wellbeing.

## 3 THE STUDY

### 3.1 Aims

Primary aim: This study aims to explore how GPNs working in general practices in England experience implementing change at pace and scale in delivering care during consecutive waves of the COVID‐19 pandemic.

Secondary Aim: To evaluate the impact of changes to general practice nurses' working practices on their professional wellbeing.

More specifically, we aim to:

- Identify changes to care delivery by GPNs during consecutive waves of the COVID‐19 pandemic.
- Identify barriers and facilitators to care delivery.
- Explore access to, and experience of translating into practice, rapidly developed guidance/evidence‐base, brought about as a response to the Covid‐19 pandemic.
- Explore acceptability of new models of working.
- Explore effects of the development of rapid and significant workforce practices on the welfare and working conditions of GPNs.

### 3.2 Design/Methodology

#### 3.2.1 Study design

Using a qualitative case study approach, data will be collected from three to five general practices in England to gain a broad perspective of how GPNs enact, experience and perceive their roles during the COVID‐19 pandemic. This will involve holding focus groups/individual interviews with GPNs and a range of primary healthcare team members, as well as key informants such as Primary Care Network leaders and general practice service commissioners (individual interviews may be conducted if it is a participant's preference or they are unable to participate in focus groups). Attendance (in‐person or remote, dependent on participants' choice and contemporary pandemic guidance) at meetings such as GPN team meetings and relevant general practice business or strategy meetings will allow observation of discussions relating to the study aims. Documents such as practice protocols and local and national policy guidelines will be analysed. At case sites, routinely collected practice data (e.g. percentage of GPN and GP consultations face‐to‐face/telephone/video/email before, during and after COVID waves) will be examined descriptively to identify changes in care delivery related to the pandemic and will be used to contextualize qualitative data. The study is funded for a 12‐month period and data are expected to be collected over a 6‐month period in this time.

Qualitative research's strengths are in gaining in‐depth understanding of a particular context, exploring underpinning meanings and unpicking how experiences, interactions and behaviours are constructed (Pope & Mays, 2006). Because of these characteristics, qualitative research has a range of applications in applied healthcare research and the study of healthcare organizations (Bowling, 2014). Consequently, this approach will be taken to gain in‐depth understanding of the effects of changing working practices during the COVID‐19 pandemic on GPNs and the study will be underpinned by a social constructivist perspective. Triangulation will be used to support comprehensiveness and consistency through the investigation of GPN working practices from a variety of perspectives (Holloway, 2008). That is we will compare several methods of data collection (observations, interviews/focus groups and document analysis) and will use more than one data source (several research sites and informants from different professional groups).

#### 3.2.2 Theoretical framework

We will explore new models of working including technology‐supported forms of working and so the study will be underpinned by the Non‐adoption, Abandonment, Scale‐up, Spread and Sustainability (NASSS) Framework (Greenhalgh et al., 2017). This is an evidence‐based, theory‐informed pragmatic framework that was originally developed to predict and evaluate success of technology‐supported healthcare. The framework's authors indicate it is not a checklist, but rather its strength lies in its adaptability to different cases and settings. NASSS has been extended to theorize and analyse service innovation and delivery more broadly in that it provides a complexity‐informed approach in which to situate implementation of new ways of working beyond a direct technology focus (Hollick et al., 2019). NASS identifies seven domains of influence which can be used to explore the acceptability and success of introducing healthcare technologies/changes in service delivery:

1. Condition: complexity of patients' illness/condition.
2. Technology: includes features/usability of technology, user needs, sustainability.
3. Value proposition: considers whether technology/service delivery is beneficial to supplier and user.
4. Adopter system: includes user engagement.
5. Organization: includes capacity, readiness, disruption and work involved in implementation at an organizational level.
6. Wider system: includes assessing impact of wider political/policy/fiscal/legal/professional/socio‐cultural contexts on implementation.
7. Embedding and adaptation over time: includes feasibility of maintaining new technology long‐term, adaption of staff roles and organizational resilience.

In this study, the NASS framework will be used to inform data generation and will allow exploration of technology‐enhanced ways of working, such as remote consultations, as well as the broader rapid adaptation of GPNs to new and different ways of delivering healthcare, while also maintaining established aspects of care which continue to be required.

#### 3.2.3 Sampling and recruitment

The researchers' university has an established network of primary care stakeholders through research and education provision. We will work with them to publicize recruitment to the study of both prospective general practice case sites and GPNs. The National Institute for Health Research Clinical Research Network may also be used to facilitate recruitment. Case sites will be purposively recruited for maximum variation [practice size, population demographics, location e.g. rural/suburban/inner city] (Braun & Clarke, 2019). Focus groups/interviews will be held with GPNs and key informants such as practice managers, GPs, administration/reception team members, Primary Care Network leaders and service commissioners, to gain a wider perspective on the role of GPNs during consecutive waves of the pandemic. Informants will be purposively sampled based on a range of roles, knowledge and insight relevant to study aims and objectives (Bowling, 2014), and invited to take part. GPNs will be purposively identified based on variation, for example experience; gender; age; role; professional level (e.g. registered nurse/advanced nurse practitioner/health care assistant/nursing associate). A snowballing strategy will also be employed with participants asked to identify other key informants.

The sampling strategy will be pragmatic, focusing on a balance between breadth and depth of data. This will enable the sample size and data generated to be manageable and comprehensive (Pope & Mays, 2006). We will not aim for saturation but instead will focus on ensuring a varied sample is represented (Braun & Clarke, 2019). It is anticipated approximately three to five case sites consisting of a total of approximately 20 GPNs plus five to eight other key informants per site will provide appropriate depth and breadth of data and is consistent with current qualitative guidance (Baker et al., 2012; Braun & Clarke, 2019).

#### 3.2.4 Data generation

Focus groups and semi‐structured interviews with participants will be informed by a topic guide developed by the research team using a priori concepts based on an adapted NASSS framework (Greenhalgh et al., 2017), research aims and current literature. For example, Turner et al. (2021) identified unintended consequences of remote consultations such as isolation and dissatisfaction of those working in general practice. However, participants did not include GPNs and it will be of value to explore whether GPNs experienced similar or divergent perspectives. Questions will centre around, for example: how and to what extent GPN practice has changed?; impact of changes on long‐term condition management?; how have changes to secondary care impacted workload (e.g. increased minor injuries presentations/blood tests?); effects of changes on workload?; training and support provision? Key ideas generated will be iteratively integrated throughout data collection into the topic guide, which will be adapted to allow contextual cross‐site comparison and contrast. Key meetings, such as GPN team meetings and business/strategy meetings will be observed and documented in field notes underpinned by research aims and NASSS framework (Greenhalgh et al., 2017).

Each case site will conduct IT searches on routinely collected data for the purposes of informing qualitative data collection. For example, we will find out the percentage of remote GPN consultations pre‐pandemic, compared with current working practices. We will also compare this to general practitioners' pre‐pandemic and current working practices. We will use these data descriptively to illustrate patterns of working of professional groups in and across case sites. We will also use these data to inform and generate focus group/interview discussion. For example, we will ask participants to comment on any differences in working practices between professional groups and explore reasons for any differences. Data generated from these discussions may then inform strategies for collecting additional descriptive practice data, that is focus group/interview discussion may provide guidance about other aspects of working practice which may be useful to explore. At each case site, general practice, Primary Care Network [PCN] and other local guidance/policy documents relating to the pandemic and practice organization will be collected, along with national guidance. For example, we will collect Royal Collage of Nursing and Queen's Nursing Institute guidance for GPNs, case site practices' protocols for telephone/remote appointments and local prescribing policies. These will again be used to generate discussion and inform topic guides as well as situate each case site in local and national contexts.

Focus groups/interviews will be conducted at a time convenient for participants, either in‐person or via telephone, Zoom (or other online video platform), dependent on interviewee preferences and contemporaneous pandemic guidance. Interviews are expected to last approximately 30–60 min and focus groups approximately 60 min. Data will be recorded and transcribed verbatim. Participants may be contacted (with permission) to seek clarity/follow‐up if necessary. Pseudonymity and confidentiality will be protected by allocating unique identifying numbers to participants and case sites.

#### 3.2.5 Researcher characteristics and reflexivity

HA's professional background is as a GPN and advanced nurse practitioner in general practice. While shared professional identity may aid rapport, it might also be anticipated that participants may make assumptions about the researcher's views of general practice, which may influence decisions around sharing information. Consequently, the potential impact on participants will be considered (Hammersley & Atkinson, 2007). HA's underlying views of nursing and its position in healthcare may be informed by socialization in nursing, general practice culture and the wider healthcare context. It is therefore important to reflexively and critically challenge views throughout the study and the context in which data are generated and analysed will be considered. This will be achieved by exploring alternative explanations and seeking disconfirming cases. Regular research team meetings will be held with PG and JA. PG is a registered nurse with a secondary care background who works in nurse education and JA is a health services researcher who does not have a clinical background. Both have significant experience of health research and neither will be directly involved in data collection or analysis. Consequently, it is anticipated that research team meeting discussions will facilitate reflection and questioning of analytical ideas.

#### 3.2.6 Data analysis

Data from focus groups/interviews, observations and documents will be analysed thematically based on the framework approach described by Pope et al. (2000). This consists of: familiarization with the data; developing a thematic framework; indexing (coding) the data; charting; mapping and interpretation. This approach is grounded in the raw data and is both informed by study aims and objectives and a priori conceptualisations. Data from different elements of the study (focus group/interview transcripts, observational fieldnotes and documents) will initially be analysed separately and then integrated in each case. They will then be compared and contrasted across case sites. Coding will be underpinned according to a priori concepts of an adapted NASSS framework (Greenhalgh et al., 2017). Data will be analysed using a constant comparative approach, with data collected and analysed concurrently. This enables developing themes and potential relationships to be tested. Interpretation of data may be influenced by the personal and professional attributes/experiences of the researcher. Consequently, a reflexive approach will be taken and a diary will be used to support reflexivity, to document analytical processes and to create an audit trail of the decisions taken and the processes of analysis.

### 3.3 Ethical considerations

The study received a favourable review from the University of York's Research Governance Committee in July 2021. As the study is an NHS workforce study, NHS Research Ethics Committee approval is not required. Health Research Authority approval has been obtained prior to the study commencing [IRAS: 30353. Protocol number: R23982. Ref 21/HRA/5132. CPMS: 51834] and formal local approval has been obtained.

Approaching and consenting participants will be conducted according to the university's research governance and NIHR Good Clinical Practice guidelines. Information sheets will explain the nature of the research, what the research involves and benefits, risks and burdens of the study to the participants. Information sheets and consent forms will advise participants that they can withdraw from the study at any point, without giving a reason and an explanation of the withdrawal procedure is detailed. Informed consent will be obtained by the lead researcher [HA], who will be conducting interviews/focus groups and observing meetings. This will be obtained through a written consent form, or via GoogleDocs if undertaken online, as per university and departmental guidelines. Study data will be obtained and managed in accordance with General Data Protection Regulations (GDPR), the Data Protection Act (2018) and university policies. Copies of consent forms and information leaflets will be given to each participant.

As a workforce study of GPNs in general practices, the study is not considered to be ethically contentious. However, qualitative studies focus on a small number of participants, as well as in‐depth contextual detail. This may, therefore, impact on protecting participants' identities. Because of this, information will be presented at a level which limits potential for identification of participants and sites.

There is a potential for inconvenience for participants and/or practices given the nature of the study. This will be minimized through careful communication and negotiation with the individuals and organizations involved. As the study is taking place in participants' workplaces (or remotely), it is not anticipated that any expenses will be accrued, and no reimbursement will be necessary. No incentives will be offered to either practices or participants.

There is a potential for the study to reveal issues about working practices and behaviours and this is made clear in the study information. Although the study is considered unlikely to reveal information requiring disclosure, because it is related to working practices, there is a potential for inappropriate or concerning behaviour to be revealed. In this event, the individual will be informed that a concern will be raised and it will be reported through the general practice's procedures for raising and escalating concerns.

### 3.4 Validity and reliability/Rigour

Quality in qualitative studies can be established through consistency between findings and broader knowledge, recognisability and relevance of findings to others, including those in similar settings, and through reflexivity (Hammersley, 1998). Comparing and contrasting similar findings in the literature contributes to the authenticity of study findings, while credibility can be assessed through transparency, reflexivity and accurate documentation of the research process. The Standards for Reporting Qualitative Research (O'Brien et al., 2014) will be used to support the quality of reporting findings from this study.

## 4 DISCUSSION

The nature of this study necessitates that it is conducted during an ongoing pandemic at a time when general practice is under severe pressure. As a consequence, the study has been planned to:

- Minimize disruption to general practices and participants by using routinely collected practice data and offering flexibility in terms of remote data collection and information provision.
- Offer flexibility to participants re whether focus groups or individual interviews are preferred and offering to attend meetings remotely if preferred by sites.
- Depth of data collection will be balanced with pressures on participating practices and participants. It is expected data collection will take place over 6 months.
- The safety of the researcher also needs to be considered and the study design allows flexibility in terms of the need for the researcher to attend in‐person or remotely.

### 4.1 Limitations

We recognize patient experience is important and funding does not allow direct evaluation of this in the current study. However, as the GPN workforce is central to primary healthcare delivery, potential issues faced by GPNs in the pandemic era have the potential to impact patient care. Consequently, this study will identify aspects of workforce practices brought about by the pandemic to inform and enhance future practice, GPN education and to inform patient care. Identifying factors which may support the GPN workforce has the potential to positively influence patient care.

Qualitative studies are based on information‐rich data, which allows deep understanding of complex research questions (Bowling, 2014). While not necessarily directly applicable, this knowledge can be transferred to other situations and contexts. This theoretical generalisability and transferability of findings can be achieved through thick description, linking findings to established and developing theories, comparison to previous work and developing evidence which resonates with the reader's existing experiential knowledge (Holloway, 2008). While experiences of individuals and general practices involved in this study may not be directly applicable to other workforces, findings may resonate with similar institutions and workforces. Furthermore, using the NASSS framework (Greenhalgh et al., 2017), may allow organizations to recognize key factors which they may choose to use to provide support for their GPN workforce.

## 5 CONCLUSION

It is anticipated this study will identify and explore aspects of patient care and workforce practices brought about by the pandemic to inform and enhance future practice, GPN education and to inform patient care. It is also intended to highlight potential difficulties and negative aspects of implementation. Key factors for success in implementing and supporting different ways of working for GPNs will be identified and working practices and systems which develop and streamline care delivery, improve patient care and support the wellbeing of the primary care workforce will be highlighted.

## CONFLICT OF INTEREST

No conflict of interest has been declared by the authors.

## AUTHOR CONTRIBUTION

All authors meet all four authorship criteria as set out in the author guidelines.

### PEER REVIEW

The peer review history for this article is available at https://publons.com/publon/10.1111/jan.15312.

## Data Availability

Data sharing not applicable to this article as no datasets were generated or analysed during the current study as it is a protocol paper
